# Subxiphoid uniportal video‐assisted thoracoscopic surgery: A cosmetically superior approach to submammary rib tumor resection

**DOI:** 10.1111/1759-7714.13218

**Published:** 2019-10-30

**Authors:** Meng Luo, Demiao Kong

**Affiliations:** ^1^ Department of Thoracic Surgery Guizhou Provincial People's Hospital Guiyang China

**Keywords:** Costectomy, fibrous dysplasia, rib tumor, subxiphoid uniportal video‐assisted thoracoscopic surgery

## Abstract

This report describes a cosmetically superior approach to submammary rib tumor resection. Surgical resection is the most effective method for the treatment of rib tumors. Common surgical methods include thoracotomy and thoracoscopic surgery. Subxiphoid uniportal video‐assisted thoracoscopic surgery (VATS) has recently been described and is being increasingly used in a variety of thoracic procedures, including thymectomy, lobectomy, and resection of giant pleural fibroids. However, there has been no report in the literature which has described the use of uniportal subxiphoid VATS for rib tumor resection. We herein report the successful removal of fibrous dysplasia of the anterolateral segment of the sixth rib by subxiphoid uniportal VATS.

## Introduction

With the development of medical devices and the promotion of minimally invasive concepts, thoracoscopic surgery has been gradually applied to the treatment of various thoracic diseases such as lung cancer,^1^ esophageal cancer,^2^, ^3^ and rib tumors.^4^ Although thoracoscopic surgery has been used for rib tumor resection during the past decade, previous reports describe single‐port or multiport thoracoscopic surgery through the lateral chest wall. The present report describes a case of subxiphoid uniportal video‐assisted thoracoscopic surgery (VATS) for left sixth rib tumor resection. The subxiphoid single‐port VATS approach was adopted because it is ideally suited to preserving cosmesis for submammary rib resection in young female patients. To the best of our knowledge, this is the first report of subxiphoid uniportal VATS costectomy.

## Case report

**Figure 1:**
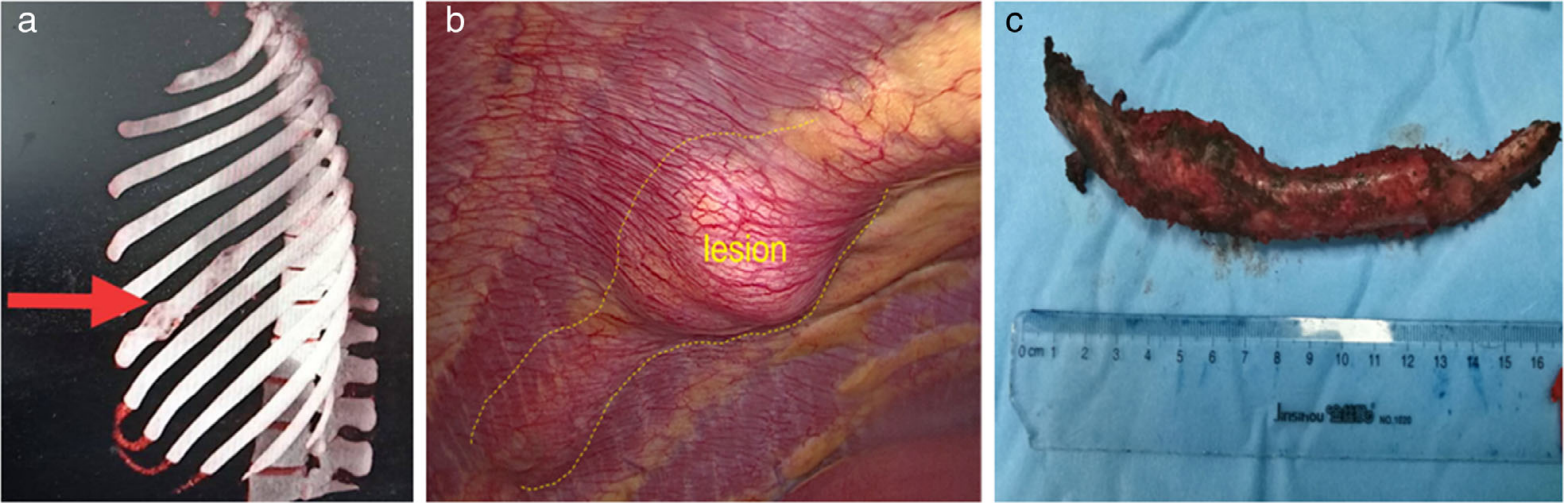
(a) Chest computed tomography image demonstrating the rib lesion. (b) Thoracoscopic view of the rib lesion. (c) The resected specimen.

A 26‐year‐old female nonsmoker was admitted to the Guizhou Provincial People's Hospital with a two‐month history of left chest wall pain. She had no significant medical history or comorbidities. Chest computed tomography revealed a 4.0 × 12.0 cm lesion with multiple foci of expansive low‐density bone destruction arising from the anterolateral segment of the sixth rib (Fig 1a). Core biopsy of the lesion showed fibrous dysplasia. The lesion was located in the sixth rib, beneath the left breast; thus, subxiphoid uniportal VATS costectomy was employed to treat the lesion and avoid damage to the breast. Informed consent was obtained from the patient for the procedure as well as for publication of the obtained clinical information.

**Figure 2:**
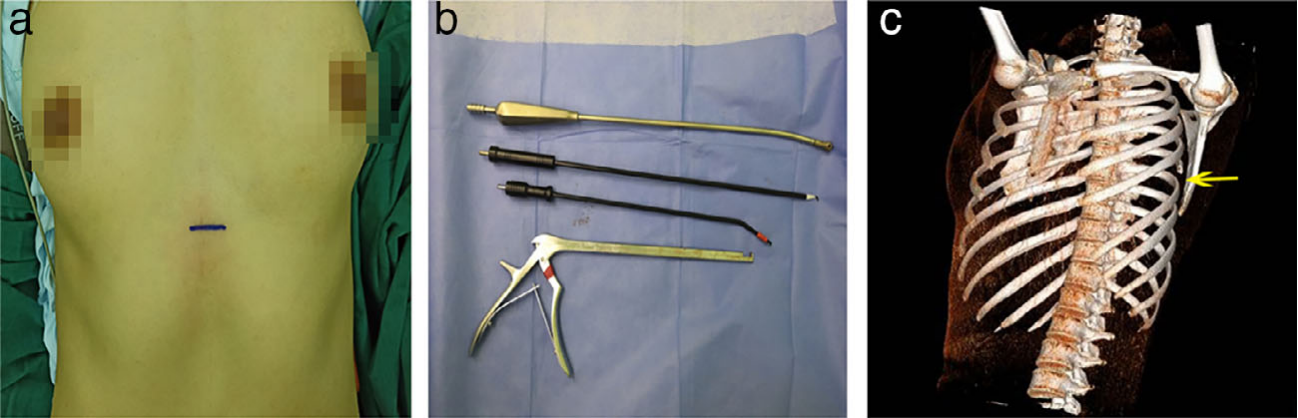
Intraoperative photographs. (a) Subxiphoid incision. (b) Instruments used to perform uniportal subxiphoid video‐assisted thoracic costectomy. (c) Postoperative chest computed tomography image.

**Figure 3:**
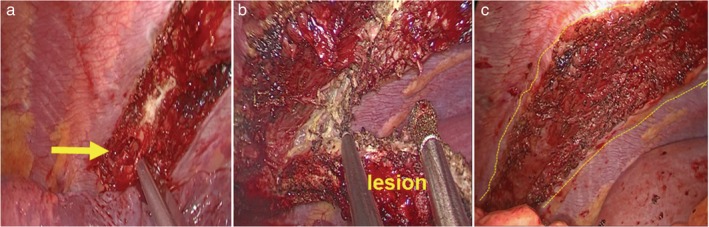
Intraoperative photographs demonstrating the visualization achieved during subxiphoid videoassisted thoracic surgery. (a) The left sixth rib was traversed with a rongeur. (b) Dissection of the left sixth rib lesion. (c) Operative wound.

The operation was performed with the patient under general anesthesia using a double‐lumen endotracheal tube to allow for selective lung ventilation. The patient was placed in the supine position with her legs spread. The scopist stood between the patient's legs and the operator stood to the right of the patient to ensure optimal visualization of the operative field. A 3.0 cm transverse incision was made below the xiphoid process (Fig 2a). The posterior surface of the sternum was blindly separated with the right index finger. The lower end of the sternum was lifted by a hanging hook, and a wound protector was placed in the incision. The left mediastinal pleura was cut, and the pericardial fat tissue was removed to optimize visualization. The left sixth rib lesion was clearly seen through the thoracoscope via selective right lung ventilation (Fig 1b). Using electrocautery, both intercostal muscles were separated from the rib, and the rib mass was transected by a bone rongeur with 3 cm margins on both sides (Fig 3a). Electrocautery was used to dissect the rib lesion while a long suction device was used to Pull the rib to optimize visualization (Fig 3b). The rib mass was removed through the subxiphoid port (Fig 1c). A 21‐Fr drain was inserted into the left thoracic cavity through the incision. The entire surgical procedure took 45 minutes to complete. Blood loss was minimal. The chest drain was removed on postoperative day 2. The final pathologic report confirmed fibrous dysplasia. No complications occurred during or after the operation, and the patient was discharged on postoperative day 4. There were no signs of recurrence six months after the operation.

## Discussion

Fibrous dysplasia is a benign lesion with a malignant transformation rate ranging from 0.4% to 4.0%.^5^ Surgical resection is an effective method for the treatment of fibrous dysplasia. A conventional approach to rib tumor resection would involve an incision greater than the length of the mass itself. Such an approach would require significant resection of the chest wall musculature and could lead to substantial postoperative morbidity and decreased quality of life.^4^ Following the widespread popularity and common use of video‐thoracoscopy in almost all types of surgical approaches ranging from complex anatomical lung resections to pleural biopsies, some surgeons^6^ have performed chest wall and rib resection by video‐assisted thoracoscope through the lateral chest approach. The lateral chest approach is a minimally invasive approach that offers several potential advantages over traditional open techniques, including decreased surgical morbidity to the chest wall musculature, decreased blood loss, and more precise localization of the ribs. However, via intercostal spaces, it is associated with intercostal nerve damage that causes numbness and pain which can persist for several months.

Subxiphoid uniportal VATS has been recently described and is being increasingly used in a variety of thoracic procedures, including thymectomy,^7^ lobectomy,^1^ and resection of giant pleural fibroids.^8^ Because this method does not require sternotomy, it is associated with lesser pain because there is no intercostal nerve damage and provides excellent cosmesis as only a single 3 cm abdominal skin incision is made, which is of great advantage to patients. However, no previous report has described the use of subxiphoid uniportal VATS for rib tumor resection. We have herein described the successful use of subxiphoid uniportal VATS to remove a benign tumor from the left sixth rib. Whilst we are of the opinion that this resection technique is especially suitable for rib tumors beneath the breast or scapula; it is not recommended for benign rib tumors above the second rib and below the seventh rib, particularly in the posterior and anterior segments of these ribs, because a good surgical field is not obtained and the surgical procedure is difficult because of interference from the heart and diaphragm.

In conclusion, subxiphoid uniportal VATS is safe, efficient, and feasible and provides good clinical outcomes for select patients with benign rib lesions.

## Disclosure

The authors report no conflicts of interest.
